# FALCON2: a web server for high-quality prediction of protein tertiary structures

**DOI:** 10.1186/s12859-021-04353-8

**Published:** 2021-09-15

**Authors:** Lupeng Kong, Fusong Ju, Haicang Zhang, Shiwei Sun, Dongbo Bu

**Affiliations:** 1 Key Lab of Intelligent Information Processing, Big-Data Academy, Institute of Computing Technology, Chinese Academy of Sciences, 100190 Beijing, China; 2 University of Chinese Academy of Sciences, 100049 Beijing, China

**Keywords:** Protein structure prediction, Template-based modeling, Ab initio prediction, Protein structure prediction web service

## Abstract

**Background:**

Accurate prediction of protein tertiary structures is highly desired as the knowledge of protein structures provides invaluable insights into protein functions. We have designed two approaches to protein structure prediction, including a template-based modeling approach (called ProALIGN) and an ab initio prediction approach (called ProFOLD). Briefly speaking, ProALIGN aligns a target protein with templates through exploiting the patterns of context-specific alignment motifs and then builds the final structure with reference to the homologous templates. In contrast, ProFOLD uses an end-to-end neural network to estimate inter-residue distances of target proteins and builds structures that satisfy these distance constraints. These two approaches emphasize different characteristics of target proteins: ProALIGN exploits structure information of homologous templates of target proteins while ProFOLD exploits the co-evolutionary information carried by homologous protein sequences. Recent progress has shown that the combination of template-based modeling and ab initio approaches is promising.

**Results:**

In the study, we present FALCON2, a web server that integrates ProALIGN and ProFOLD to provide high-quality protein structure prediction service. For a target protein, FALCON2 executes ProALIGN and ProFOLD simultaneously to predict possible structures and selects the most likely one as the final prediction result. We evaluated FALCON2 on widely-used benchmarks, including 104 CASP13 (the 13th Critical Assessment of protein Structure Prediction) targets and 91 CASP14 targets. In-depth examination suggests that when high-quality templates are available, ProALIGN is superior to ProFOLD and in other cases, ProFOLD shows better performance. By integrating these two approaches with different emphasis, FALCON2 server outperforms the two individual approaches and also achieves state-of-the-art performance compared with existing approaches.

**Conclusions:**

By integrating template-based modeling and ab initio approaches, FALCON2 provides an easy-to-use and high-quality protein structure prediction service for the community and we expect it to enable insights into a deep understanding of protein functions.

**Supplementary Information:**

The online version contains supplementary material available at 10.1186/s12859-021-04353-8.

## Background

Proteins are macromolecules composed of amino acid chains and serve important roles in a wide-range of biological processes including catalysis, immunity, and information transmission. A protein performs its biological functions by folding into specific tertiary structures; thus, the knowledge of protein structure is crucially helpful for the deep understanding of its biological functions [1]. Protein structures can be experimentally determined using X-ray crystallography, nuclear magnetic resonance, or cryo-electron microscopy. These experimental technologies, however, are usually time-consuming and thus cannot catch up with the rapid accumulation of protein sequences. Unlike these experimental technologies, the computational prediction of protein structures purely from amino acid sequences is efficient, and accurate prediction approaches are highly desired.

Protein structure prediction has received extensive studies and a large variety of prediction approaches have already been proposed. These approaches can be divided into two categories, namely, template-based modeling (TBM) approaches and ab initio prediction approaches. For a target protein of interest, TBM approaches first identify its homologous proteins with known structures (called *templates*) through constructing alignments, and then build tertiary structures with reference to the structure of these homologous proteins [2, 3]. Statistical models [4] and combinatorial optimization techniques [5, 6] are widely used to model and calculate the optimal protein alignment. When homologous templates of the target protein are available and high-quality target-template alignments can be constructed, TBM approaches can accurately predict structures for the target protein.

Unlike the template-based modeling approaches, ab initio prediction approaches do not require the availability of homologous templates for target proteins; instead, these approaches predict protein structures in an ab initio fashion, i.e., constructing structures with the lowest free energy [7]. For instance, Rosetta uses an energy function describing Van der Waals force, hydrophobic effects and hydrogen bonds, and uses the Monte Carlo strategy to find the structure that minimizes the energy function [7]. I-TASSER constructs high-quality structural models through iterative threading assembly refinement [8]. The past decade has witnessed a great breakthrough in ab initio prediction approaches: using the inter-residue distance derived from direct-coupling analysis of homologous protein sequences [9, 10], trRosetta [11] and AlphaFold [12] predict structures of target proteins with significantly improved accuracy. Recently, deep learning has been widely applied to improve the estimation of inter-residue distances and construct structures that satisfy the distance restrictions [13–15].

Recent advances have shown that the combination of TBM and ab initio approaches is promising as these two types of approaches have different emphasis [16, 17]. Generally speaking, the TBM approaches exploit structure information of homologous templates of target proteins while the recent ab initio approaches usually exploit the co-evolutionary information carried by homologous proteins. We have designed two approaches to protein structure prediction, including a template-based modeling approach ProALIGN [18] and an ab initio prediction approach ProFOLD [19]. Specifically, ProALIGN uses a deep neural network to learn the patterns of context-specific alignment motifs. These patterns enable ProALIGN to model the dependence among residue pairs and thereafter accurately construct target-template alignments for structure building. Unlike the existing approaches using handcrafted features such as covariance matrix [14, 17], ProFOLD employs an end-to-end framework called CopulaNet to estimate inter-residue distances directly from multiple sequence alignment (MSA) of the target protein.

In the study, we present the FALCON2 server that integrates the TBM approach ProALIGN and ab initio approach ProFOLD. For a target protein, we run these two approaches to predict tertiary structures simultaneously, then employs a quality assessment tool ProQ3D to estimate structure quality, and finally selects the best candidate structures from the prediction results by the two approaches. Using 104 CASP13 targets and 91 CASP14 targets, we evaluated FALCON2 server and performed a systematic analysis and comparison of these two approaches. These experimental results suggest that by integrating TBM and ab initio approaches, FALCON2 can predict protein structures with improved accuracy and efficiency. FALCON2 also has a user-friendly interface and we expect it to enable insights into a deep understanding of protein functions.

## Implementation

**Fig. 1:**
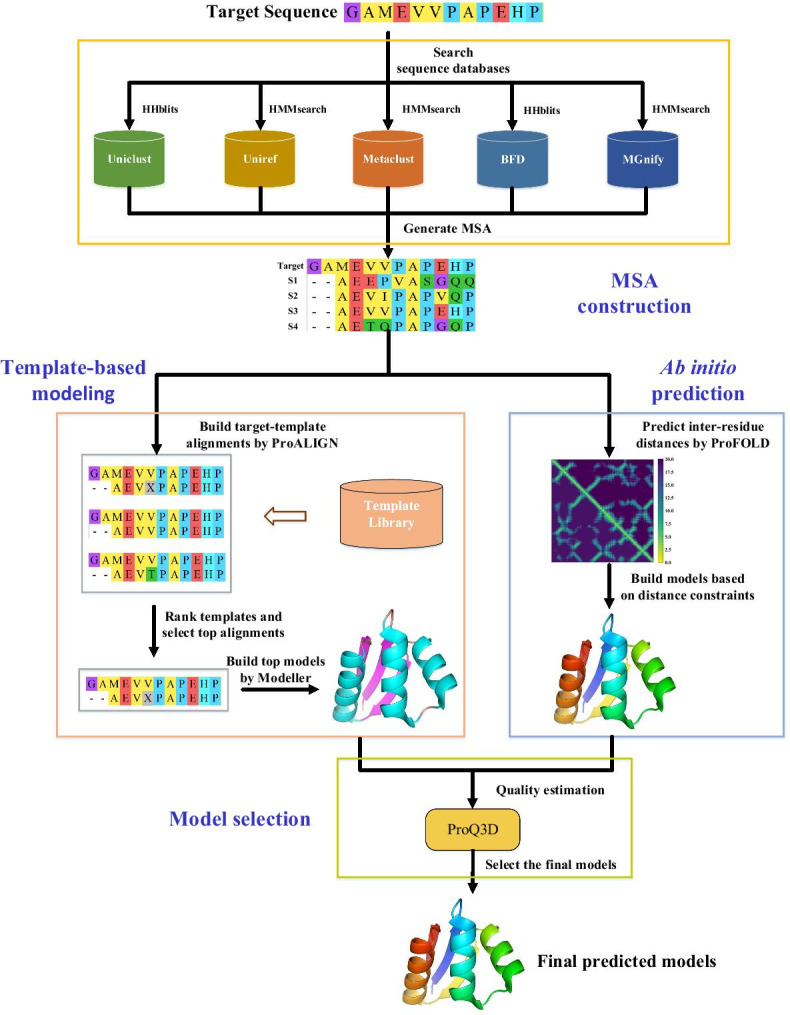
Workflow of FALCON2 prediction server. The prediction procedure of FALCON2 mainly consists of the following four steps: (i) Constructing MSA for target protein: For a target protein input by user, FALCON2 first searches its homologous proteins within a variety of sequence databases including Uniclust, Uniref, Metaclust, BFD and MGnify. The identified homologous proteins are organized as a multiple sequence alignment (MSA). (ii) Predicting candidate structures using ProALIGN: For each target protein, FALCON2 predicts its candidate structures by running ProALIGN and ProFOLD simultaneously. Specifically, ProALIGN takes the constructed MSA as input and calculates target-template alignment with all templates in a pre-defined library. The library will be regularly updated. ProALIGN selects the most likely target-template alignment according to contact information, and subsequently builds candidate structures with reference to the selected templates. (iii) Predicting candidate structures using ProFOLD: ProFOLD uses an end-to-end framework, called CopulaNet, to estimate inter-residue distances directly from MSA of target protein. ProFOLD transforms the estimated inter-residue distances into an energy potential, and applies the gradient-descent technique to build candidate structures that minimize this potential function. (iv) Selecting the best candidate structure: For all the candidate structures predicted by ProALIGN and ProFOLD, FALCON2 evaluates their quality through running ProQ3D, a software for structure quality assessment. The candidate structure with the highest quality will be reported as the final prediction result by FALCON2

For a target protein, FALCON2 predicts its structure using a four-step procedure, including constructing MSA of the target protein, executing ProALIGN and ProFOLD simultaneously to yield candidate structure models, and subsequently selecting the best model as the final prediction result. The flowchart of FALCON2 is shown in Fig. 1 and more details of these four steps are described as follows.

### Constructing MSA of target protein

Construction of high-quality MSA for target protein is the first and fundamental step of the entire prediction procedure. The quality of MSA has great effects on protein alignment, inter-residue distance estimation, and structure quality assessment [20]. To build high-quality MSA, FALCON2 executes HHblits [21] and HMMsearch [22] to search target protein for its homologous proteins within Uniclust [23] and UniRef [24] sequence databases. For virus or bacterial proteins, the MSA thus constructed might have only a few homologous proteins. In this case, we further search target protein against metagenome databases including Metaclust database [25], BFD database [26], and MGnify database [27].

### Template-based modeling using ProALIGN

To predict candidate structures for the target protein, we first construct target-template alignment by running ProALIGN with the constructed MSA as input, select the most likely alignment and template, and then generate candidate structures with reference to template structure.

Unlike the existing TBM approaching using a handcrafted scoring function for alignments, ProALIGN directly learns and infer protein alignment through exploiting the patterns of context-specific alignment motifs. Specifically, ProALIGN represents an alignment as a binary matrix in which the symbol ‘1’ denotes an aligned residue pair and ‘0’ denotes unpaired residues. This representation clearly shapes alignment motifs, e.g., aligned helices are shown as diagonal lines while alignment gaps are shown as two diagonal lines with a shift between them. These alignment motifs are context-specific, thus enabling us to recognize alignment motifs based on sequence contexts. ProALIGN uses a deep convolutional neural network to learn the patterns of alignment motifs.

For each template, ProALIGN applies the neural network to directly infer likelihoods of all possible residue pairs with target protein in their entirety, and then constructs the alignment with maximum likelihood. ProALIGN ranks all templates using a CMO-style [28] scoring function and uses the top 5 templates as references to build candidate structures using Modeller [29].

### Ab initio protein structure prediction using ProFOLD

ProFOLD predicts structures for target proteins in ab initio fashion. ProFOLD uses an end-to-end framework, called CopulaNet, to estimate inter-residue distances directly from the MSA of the target protein. The CopulaNet consists of the following three key elements: (1) MSA encoder: according to each homologous protein collected in MSA, ProFOLD uses an MSA-encoder to extract context-specific mutation information of the target residues. (2) Co-evolution aggregator: ProFOLD applies a co-evolution aggregator to calculate residue co-evolution. (3) Inter-residue distances estimator: Subsequently, a distance estimator is used to estimate inter-residue distances according to the acquired residue co-evolution.

Finally, ProFOLD transforms the estimated inter-residue distances into an energy potential, and applies the gradient-descent technique to build structures that minimize this potential function [11, 30]. We run ProFOLD to generate 100 decoys and then use the top 5 decoys with the lowest energy potential as candidate structures for further selection.

### Estimating structure quality and selecting the best candidate structure

For the candidate structures predicted by ProALIGN and ProFOLD, FALCON2 estimates structure quality by running ProQ3D [31]. Briefly speaking, ProQ3D assesses the quality of a structure by considering a variety of features, including residue contacts, residue conservation, and the agreement with the predicted secondary structure and solvent accessibility area. ProQ3D also takes into consideration the energy terms calculated by Rosetta [7]. ProQ3D feeds these features into a deep neural network, thus yielding the predicted structure quality, including TM-score, GDT_TS, and lDDT. By running ProQ3D on all candidate structures, FALCON2 obtains the predicted quality value lDDT and normalizes them into Z-score. FALCON2 finally selects the candidate structure with the highest lDDT as the final prediction result.

### The user interface for FALCON2 server

FALCON2 provides an easy-to-use web service for protein structure prediction. It accepts protein sequence in FASTA format as input and returns the predicted structure of the target protein. FALCON2 also reports additional information for further analysis, including the constructed MSA, target-template alignments, predicted residue contacts, inter-residue distances, and Ramachandran plots of the predicted structures. FALCON2 provides an intuitive way to visualize the predicted 3D structures. Additional file 1: Figures S8-S13 show examples of the job submission page, job status page, and result visualization page.

## Results and discussions

We evaluated the performance of FALCON2 over CASP13 and CASP14 official-defined domain targets, and compared FALCON2 with the best CASP server groups. The prediction results by the CASP13 and CASP14 groups were downloaded from the CASP official website.

For each of the predicted structures, we superimposed it onto the corresponding native structure, calculated TM-score, and used it to measure the quality of the predicted structure. TM-score ranges from 0 to 1, and a high TM-score implies that the two protein structures under consideration are similar. It should be noted that the sequence and template databases used by FALCON2 are regularly updated; however, to make the evaluation and comparison fair, we used only the protein sequences and structures released before CASP13 and CASP14 competitions accordingly.

### The performance of FALCON2 over CASP13 targets

We first evaluated the performance of FALCON2 over 104 CASP13 official-defined domain targets, and compared FALCON2 with the best CASP13 server and human groups, including A7D, Zhang, MULTICOM, QUARK, Zhang-Server, and RaptorX-DeepModeller. For each CASP13 domain target, we constructed MSA through searching it against three sequence databases, including Uniclust30 (as of Oct. 2017), Uniref90 (as of Mar. 2018), and metagenome database Metaclust (as of Jan. 2018). We used the structures recorded in the PDB database (as of Apr. 2018) as templates to build candidate structures.

We summarized the prediction performance by FALCON2 and six CASP13 groups in Table 1. As shown in this table, over the 104 CASP13 domains, the average TM-score of the predicted protein structures by FALCON2 is 0.755, higher than the top human groups (A7D: 0.699, Zhang: 0.692, MULTICOM: 0.688) and server groups (QUARK: 0.672, Zhang-Server: 0.671, RaptorX-DeepModeller: 0.653). Specifically, for the 31 FM domains, FALCON2 achieves high prediction quality (average TM-score: 0.665), which is better than the state-of-the-art approaches (A7D: 0.580, Zhang: 0.509, MULTICOM: 0.495).

We further investigated the predicted structures by ProALIGN and ProFOLD individually and analyzed the contributions by these two components to FALCON2. Table 1 suggests that the top 1 predicted structures by ProALIGN and ProFOLD show an average TM-score of 0.644 and 0.736, respectively. By combining these two approaches, FALCON2 achieves an average TM-score of 0.755, which is higher than the two approaches.

In-depth examination suggests that the combination strategy leads to significant performance improvement, especially for the TBM target proteins. Specifically, for TBM-hard targets, the top 1 predicted structures by ProALIGN and ProFOLD show an average TM-score of 0.701 and 0.700, respectively. In contrast, FALCON2 achieves an average TM-score of 0.763.

**Table 1:** TM-score of the predicted structures for CASP13 targets by FALCON2 and top CASP13 groups

Group	All domains (104)	TBM-easy domains (40)	TBM-hard domains (21)	FM/TBM domains (13)	FM domains (31)
A7D (Human)	0.699/0.733	0.793/0.818	0.700/0.724	0.691/0.739	0.580/0.626
Zhang (Human)	0.692/0.719	**0.841/0.850**	0.724/0.750	0.605/0.665	0.509/0.549
MULTICOM (Human)	0.688/0.722	0.830/0.849	0.725/0.757	0.645/0.675	0.495/0.551
QUARK	0.672/0.699	0.823/0.840	0.714/0.748	0.589/0.648	0.479/0.503
Zhang-server	0.671/0.699	0.821/0.840	0.721/0.743	0.593/0.627	0.475/0.514
RaptorX-DeepModeller	0.653/0.674	0.820/0.832	0.687/0.697	0.561/0.592	0.451/0.486
ProALIGN-only	0.644/0.659	0.815/0.829	0.701/0.722	0.582/0.598	0.408/0.420
ProFOLD-only	0.736/0.745	0.813/0.816	0.700/0.716	0.727/0.732	0.664/0.677
FALCON2	**0.755/0.766**	0.828/0.839	**0.763/0.776**	**0.731/0.736**	**0.665/0.677**

Here, we show model quality (measured using TM-score) of the top 1 and the best of top 5 predicted structures. The best performance is marked in bold font

### The performance of FALCON2 over CASP14 targets

We also evaluated the performance of FALCON2 over 91 CASP14 official-defined domain targets, and compared FALCON2 with the top CASP14 server groups, including Zhang-Server, BAKER-ROSETTASERVER, Yang-Server, tFold, and FEIG-S. For each CASP13 domain target, we constructed MSA through searching it against four sequence databases, including Uniref30 (as of Feb. 2020), Uniref90 (as of Feb. 2020), BFD (as of Mar. 2019), and MGnify90 (as of May. 2019). We used the structures recorded in the PDB database (as of Apr. 2020) as templates to build candidate structures.

**Table 2:** TM-score of the predicted structures for CASP14 targets by FALCON2 and top CASP14 server groups

	All (91)	TBM-easy (26)	TBM-hard (28)	FM/TBM (14)	FM (23)
Zhang-server	0.706/**0.725**	0.855/0.863	0.698/**0.717**	0.694/**0.733**	0.555/**0.574**
BAKER-ROSETTASERVER	0.655/0.665	0.837/0.846	0.705/0.716	0.672/0.680	0.378/0.392
Yang-Server	0.667/0.695	0.842/0.851	0.685/0.707	0.680/0.698	0.438/0.501
tFold	0.660/0.683	0.823/0.834	0.692/0.711	0.626/0.674	0.456/0.485
RaptorX	0.652/0.676	**0.859**/**0.866**	0.692/0.731	0.620/0.638	0.388/0.417
FEIG-S	0.626/0.634	0.844/0.848	0.669/0.679	0.634/0.646	0.323/0.330
ProALIGN-only	0.566/0.584	0.805/0.823	0.621/0.635	0.437/0.465	0.307/0.323
ProFOLD-only	0.698/0.704	0.816/0.821	0.693/0.698	0.710/0.717	**0.564**/0.568
FALCON2	**0.712**/0.720	0.850/0.862	**0.708**/0.714	**0.713**/0.717	0.562/0.568

Here, we show TM-score of top1/top5 predicted models. The best performance is marked in bold font

As shown in Table 2, the average TM-score of the top 1 predicted structures by FALCON2 is 0.712, which is better than all CASP14 server groups (Zhang-Server: 0.706, BAKER-ROSETTASERVER: 0.655, Yang-Server: 0.657, tFold: 0.660). The superiority of FALCON2 is much clearer for the FM target proteins. The average TM-score of the top 1 predicted structures for FM target by FALCON2 is 0.562, which is better than Zhang-Server (0.555), and much better than the other CASP14 server groups (BAKER-ROSETTASERVER: 0.378, Yang-Server: 0.438, tFold: 0.456).

Overall, these results lead to similar observations that have been obtained on CASP13 target proteins, i.e., the combination strategy has higher prediction accuracy than the individual prediction approach, especially for the TBM target proteins.

## Analyzing the contributions by ProFOLD and ProALIGN to FALCON2

**Fig. 2:**
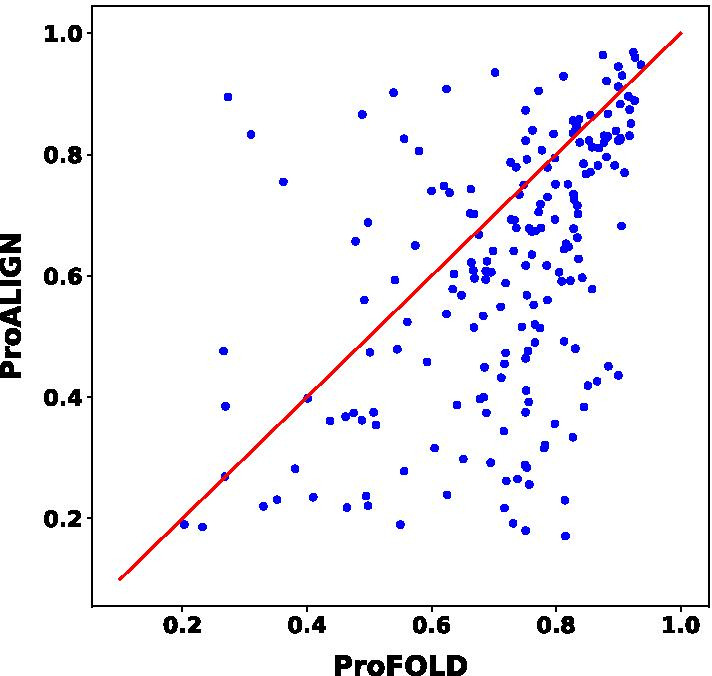
Head-to-head comparison of ProFOLD and ProALIGN on CASP13 and CASP14 targets. Each point represents two predicted structures generated by ProFOLD (*x*-axis) and ProALIGN (*y*-axis), respectively

In order to figure out the contribution of ProFOLD and ProALIGN to FALCON2, we performed the head-to-head comparison of the two approaches on CASP13 and CASP14 targets. Figure 2 shows that in general, ProFOLD has better prediction performance than ProALIGN and in some cases, the prediction structures by ProALIGN are better than ProFOLD.

To further examine in what cases ProALIGN outperforms ProFOLD, we divided the CASP13 and CASP14 target proteins into groups according to the availability of high-quality templates. In particular, for each target protein, we calculate the TM-score between its native structure and the most similar template. Next, we divide the target proteins into four groups with calculated TM-score within [0.00, 0.40], (0.40, 0.60], (0.60, 0.80], and (0.80, 1.00], respectively. As shown in Table 3, for the targets in the [0.00, 0.40], (0.40, 0.60], and (0.60, 0.80] groups, ProFOLD generates higher-quality protein structure than ProALGIN. In contrast, for the targets in (0.80, 1.00] group, ProALIGN outperforms ProFOLD. These results suggest that when high-quality templates are available, ProALIGN is superior to ProFOLD and in other cases, ProFOLD shows better performance.

**Table 3:** Quality of the predicted structures by ProALIGN, ProFOLD and FALCON2 on CASP13 and CASP14 target proteins

Template quality	#Targets	ProFOLD	ProALIGN	FALCON2
[0.00, 0.40 ]	22	**0.634**	0.252	**0.634**
(0.40, 0.60 ]	45	0.660	0.433	**0.662**
(0.60, 0.80 ]	78	0.727	0.651	**0.738**
(0.80, 1.00 ]	50	0.793	**0.854**	0.845

Here, we measure the structure similarity of template and target protein using TM-score and split all the targets into four groups: < 0.40 0.40–0.60, 0.60–0.80, and 0.80–1.00. The best performance is marked in bold font

## Case studies

Using two proteins (T0950 and T0966) as representatives, we demonstrated the details of the prediction procedure of FALCON2, including the constructed MSA, predicted inter-residue distances, the selected templates, and the constructed structure models.

### Case study 1: CASP13 target T0966

The target protein T0966 has a total of 492 residues, which was classified as TBM-hard in the CASP13 competition. The protein is a MARTX toxin effector domain from Vibrio vulnificus CMCP [32], and its native structure has already been solved and deposited in PDB as 5w6iA.

**Table 4:** Precision of the contacts predicted by ProFOLD for T0966

	Top *L*	Top *L*/2	Top *L*/5	Top *L*/10
Long-range	0.443	0.610	0.806	1.000
Medium-range	0.254	0.472	0.765	0.898
Short-range	0.262	0.471	0.878	0.980

Here, we show the precision of the top L/10, L/5, L/2 and L residue contacts, where L represents protein length

**Fig. 3:**
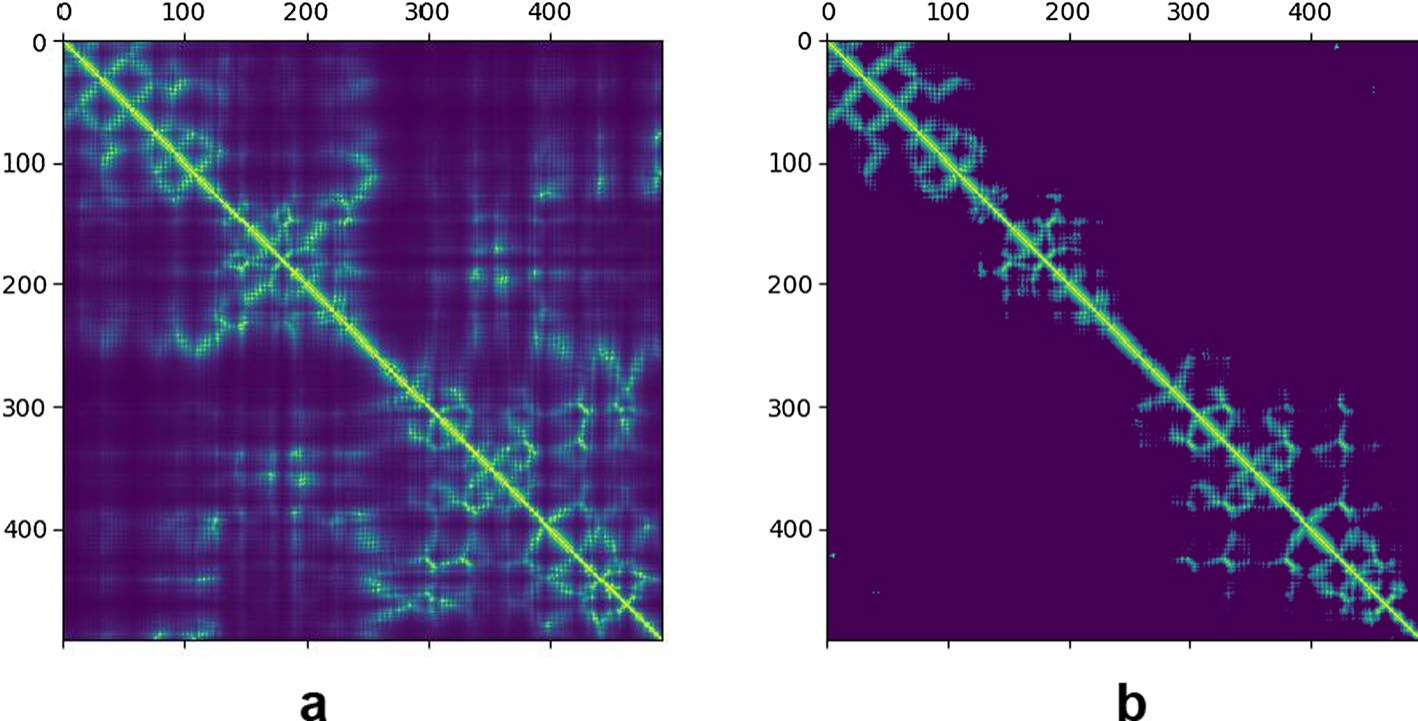
The native inter-residue distances (left panel) and the predicted inter-residue distances by ProFOLD (right panel) for target T0966

For T0966, FALCON2 constructed an MSA through searching it against three sequence databases, including Uniclust30 (as of Oct. 2017), Uniref90 (as of Mar. 2018), and Metaclust (as of Jan. 2018). The constructed MSA contains a total of 126 homologous proteins, implying that its quality is relatively lower. Next, FALCON2 executed ProFOLD to predict inter-residue distances for this target. However, due to the low-quality MSA, the accuracy of the predicted inter-residue contacts is relatively lower. As shown in Table 4, the prediction accuracy of top *L* long-range residue contacts is only 0.443. The predicted inter-residue distances also deviate significantly from the native one (Fig. 3). Consequently, the predicted structure by ProFOLD achieved a TM-score of only 0.310.

**Table 5:** The top 5 templates reported by ProALIGN for target T0966

Template	Sequence identity (%)	Confidence score	Template quality	Quality of the predicted structure
2ebhX	25.4	0.699	0.820	0.833
2ebfX	25.2	0.697	0.817	0.834
2ec5A	25.9	0.691	0.822	0.852
4r04A	11.5	0.362	0.208	0.254
4w8fA	8.60	0.361	0.195	0.213

Here, we use TM-score to measure template quality and predicted structure quality

**Table 6:** The top 5 predicted structures reported by ProQ3D for target T0966

Predicted structure	Predicted lDDT score	Z-score	True lDDT score	TM-score of the predicted structure
T0966-PA-2ebhX	0.559	1.049	0.632	1.05
T0966-PA-2ebfX	0.546	0.833	0.628	0.833
T0966-PA-2ec5A	0.524	0.480	0.626	0.852
T0966-PF-m1	0.516	0.354	0.354	0.254
T0966-PF-m2	0.514	0.315	0.315	0.213

Here, we use lDDT score and TM-score to measure predicted structure quality

**Fig. 4:**
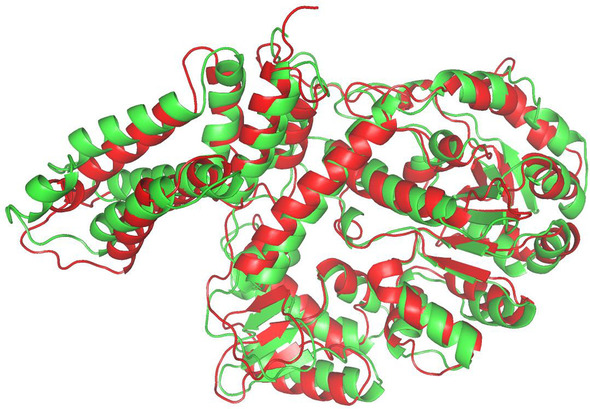
The native structure (in green) and predicted structure (in red) by FALCON2 for target T0966 (TM-score: 0.833)

For this target, FALCON2 also executed ProALIGN to yield candidate structures. Table 5 shows the top 5 templates reported by ProALIGN. In the case of template 2ebhX, its sequence identity with T0966 is 25.4%, and according to this template, ProALIGN constructed a target-template alignment with a high confidence score (0.699). In fact, this template is substantially similar to the native structure (TM-score: 0.820), and using this template to construct target-template alignment, ProALIGN yielded a high-quality structure with TM-score 0.833. Finally, according to the predicted lDDT reported by ProQ3D (Table 6), FALCON2 selected the structure predicted by ProALIGN as the final prediction result (TM-score: 0.833; Fig. 4).

### Case study 2: CASP13 target T0950

**Table 7:** Precision of the inter-residue contacts predicted by ProFOLD for target T0950

	Top L	Top L/2	Top L/5	Top L/10
Long-range	0.675	0.610	1.000	1.000
Medium-range	0.090	0.187	0.471	0.794
Short-range	0.060	0.090	0.190	0.294

Here, we show the precision of the top L/10, L/5, L/2, and L residue contacts, where L represents protein length

The target protein T0950 has a total of 353 residues, which was classified as FM in the CASP13 competition. T0950 is a membrane protein from Photorhabdus luminescens [33], and its native structure has already been solved and deposited in PDB as 6ek4A.

**Fig. 5:**
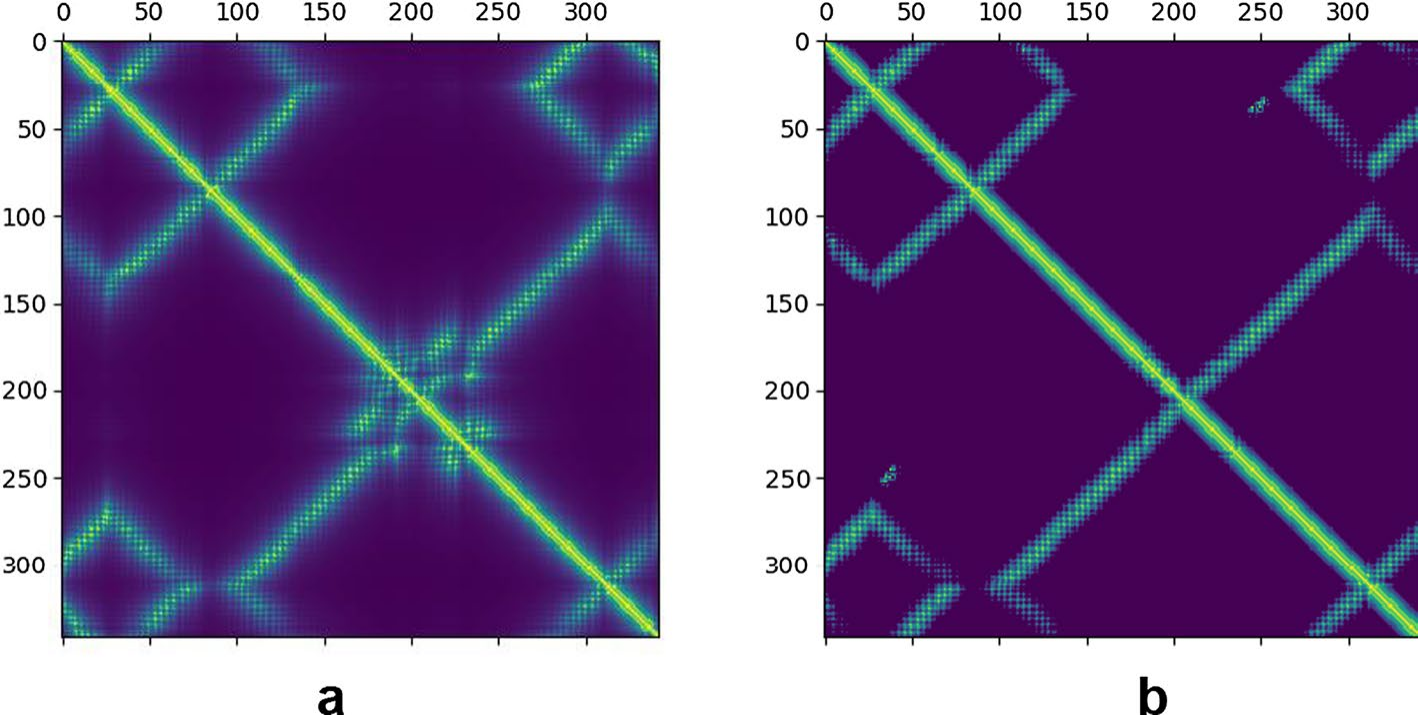
The native inter-residue distances (left panel) and the predicted inter-residue distances by ProFOLD (right panel) for target T0950

**Table 8:** The top 5 templates reported by ProALIGN for target T0950

Template	Sequence identity (%)	CMO score	Template quality	Predicted model quality
5gheA	11.1	0.500	0.335	0.372
5j66A	11.9	0.500	0.336	0.327
5j65A	12.9	0.498	0.329	0.315
5kucA	12.5	0.474	0.331	0.326
4k1pA	6.80	0.459	0.297	0.305

Here, we use TM-score to measure template quality and predicted structure quality

**Table 9:** The top 5 predicted structures reported by ProQ3D for target T0950

Predicted structure	Predicted lDDT score	Z-Score	True lDDT score	TM-score of the predicted structure
T0950-PF-m1	0.572	1.161	0.620	0.730
T0950-PA-m2	0.560	1.025	0.617	0.751
T0950-PA-m3	0.555	0.967	0.622	0.731
T0950-PF-m4	0.554	0.953	0.608	0.758
T0950-PF-m5	0.545	0.860	0.595	0.783

Here, we use lDDT score and TM-score to measure predicted structure quality

**Fig. 6:**
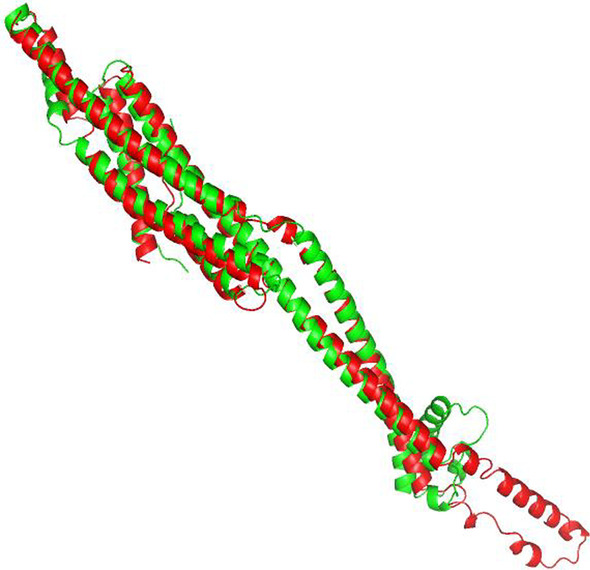
The native structure (in green) and predicted structure (in red) by FALCON2 for target T0950 (TM-score: 0.730)

For T0950, FALCON2 constructed an MSA through searching it against three sequence databases, including Uniclust30 (as of Oct. 2017), Uniref90 (as of Mar. 2018), and Metaclust (as of Jan. 2018). The constructed MSA contains a total of 462 homologous proteins, implying that its quality is relatively high. Next, FALCON2 executed ProFOLD to predict inter-residue distances for this target. Using the high-quality MSA, ProFOLD yielded accurate distance prediction. As shown in Table 7, the prediction accuracy of top *L* long-range residue contacts reaches 0.675. The predicted inter-residue distance matrix is also similar to the native one (Fig. 5). Consequently, the predicted structure by ProFOLD achieved a TM-score of 0.730.

For this target, FALCON2 also executed ProALIGN to yield candidate structures. Table 8 shows the top 5 templates reported by ProALIGN. As no homologous template has been deposited in the template database used in this study, ProALIGN failed to find a similar template and thus cannot generate high-quality structure models. Finally, according to the predicted lDDT reported by ProQ3D (Table 9), FALCON2 selected a structure predicted by ProFOLD as the final prediction result (TM-score: 0.730; Fig. 6).

## Conclusion

In this study, we present FALCON2, a web server for high-quality protein structure prediction. Using CASP13 and CASP14 target proteins as representatives, we demonstrate that FALCON2 can successfully predict structures for both TBM and FM target proteins when high-quality MSA can be obtained. We also observed that TBM and ab initio approaches have different emphasis, and the combination of these two types of approaches can lead to improved prediction accuracy. FALCON2 provides a user-friendly graphic interface, making it easy to use for the community. We expect FALCON2 web service to enable insights into the structure and function of proteins, especially the proteins with important roles in health and disease.

### Availability and requirements

Project name: FALCON2 server

Project home page: http://protein.ict.ac.cn/FALCON2

Operating system(s): Windows, Linux, Mac

Programming language: Python, PHP, C++

License: GPL

Any restrictions to use by non-academics: license needed

## Supplementary Information

**Additional file 1**. Files containing additional implementation detail and additional tables of results.

## Data Availability

The FALCON2 web server is freely available online at http://protein.ict.ac.cn/FALCON2. The data generated and analyzed during the current study are available at http://protein.ict.ac.cn/FALCON2/experiment_data/falcon2data.tgz.

## Abbreviations

TBM: Template-based modeling method
CASP: Critical assessment of protein structure prediction
MSA: Multiple sequence alignment
lDDT: local Distance Difference Test
PSSM: Position specific scoring matrix
