# Supplementary material for: FALCON2: a web server for high-quality prediction of protein tertiary structures
Source: BMC Bioinformatics. 2021 Sep 15;22:439. doi: 10.1186/s12859-021-04353-8 (PMC8444573; doi:10.1186/s12859-021-04353-8)
Supplement: Supplementary file 1 — Additional file 1. Files containing additional implementation detail and additional tables of results. [file 12859_2021_4353_MOESM1_ESM.pdf]

Supplementary Material
FALCON2: a web server for high-quality prediction of protein tertiary structures

Lupeng Kong[a,b,†], Fusong Ju[a,b,†], Shiwei Sun[a,b], Haicang Zhang[a,b] and Dongbo Bu *[a,b]

[a] *Key Lab of Intelligent Information Processing, Big-data Academy, Institute of Computing Technology, Chinese Academy of Sciences, Beijing 100190, China*
[b] *University of Chinese Academy of Sciences, Beijing 100049, China*

Contents

1 Prediction performance of FALCON2 at the fold level 2

2 Reliability of the quality assessment of the predicted structures 2

3 The effects of the availability of similar templates on the final prediction performance 4

4 Performance of FALCON2 on the combined CASP13 and CASP14 datasets 5

5 Two case studies: all-beta protein T0965 and alpha/beta protein T1026 5
5.1 All-beta protein: CASP13 target T0965 6
5.2 Alpha/beta protein: CASP14 target T1026 8

6 The user interface of FALCON2 server and usage explanation 10
6.1 The job submission interface of FALCON2 server 10
6.2 The running status interface of FALCON2 server 11
6.3 Result display interface of FALCON2 server 12

*All correspondence should be addressed to Dongbo Bu (dbu@ict.ac.cn). The first two authors contributed equally to the study.

1 Prediction performance of FALCON2 at the fold level

In addition to evaluating prediction accuracy in terms of TM-score, we also evaluated the prediction performance of FALCON2 at the fold level. Previous studies have shown that if TM-score exceeds 0.50, the two protein structures are generally in the same fold topology, while TM-score less than 0.2 means no structural similarity between the two protein structures [4].

Table S1 shows the prediction performance of FALCON2 at the fold topology level on the CASP13 dataset. As shown in this table, for 97 out of 104 CASP13 target proteins, the predicted structures achieved TM-score over 0.50, which implies that the percentage of the protein structures with correctly-predicted fold topology is as high as 93.3%. The percentage further increases to 96.2% when considering the best of top 5 predicted models.

Table S2 suggests that for 77 out of the 91 CASP14 targets, the predicted structures achieved TM-score exceeding 0.5, implying that the percentage of the predicted structures with correct fold topology is 84.6%. The percentage reaches 87.9% when considering the best of top 5 predicted models.

In summary, these results demonstrate that FALCON2 can accurately predict protein fold topology.

Table S1: The percentage of predicted structures with correct fold topology. Dataset: CASP13 domains

	All (104)	TBM-easy (40)	TBM-hard (21)	FM/TBM (12)	FM (31)
A7D (Human)	89/91	38/38	19/19	11/11	21/23
Zhang (Human)	86/91	**40/40**	18/20	10/11	18/20
MULTICOM (Human)	86/93	**40/40**	19/21	11/11	16/21
QUARK	84/88	**40/40**	19/20	10/11	15/17
Zhang-Server	84/90	**40/40**	19/20	10/11	15/19
RaptorX-DeepModeller	83/84	**40/40**	19/19	9/9	15/16
FALCON2	**97/100**	39/**40**	**21/21**	**12/12**	**26/27**

Here, we show results of top 1 and the best of top 5 predicted structures. The best performance is marked in bold font.

2 Reliability of the quality assessment of the predicted structures

In FALCON2, we use the predicted lDDT reported by ProQ3D to assess the quality of the predicted structures. To analyze the reliability of the predicted lDDT, we calculated the correlation between the predicted lDDT score and the true lDDT score. As shown in Figure S1, the predicted lDDT has strong correlation with true lDDT (Pearson correlation coefficient: 0.84), which suggests the reliability of the predicted lDDT.

Table S2: Percentage of the predicted structures with the correct fold topology. Dataset: CASP14 target proteins

	All (91)	TBM-easy (26)	TBM-hard (28)	FM/TBM (14)	FM (23)
Zhang-Server	75/78	**26**/**26**	25/26	12/**13**	**12**/13
BAKER-ROSETTASERVER	67/68	25/**26**	25/25	12/12	5/5
Yang-Server	74/78	**26**/**26**	27/27	12/**13**	9/12
tFold	67/74	**26**/**26**	24/26	10/13	7/9
RaptorX	68/71	**26**/**26**	**27**/**27**	11/12	4/6
FEIG-S	63/64	**26**/**26**	24/24	11/12	2/2
FALCON2	**77**/**80**	**26**/**26**	26/26	**13**/**13**	**12**/**15**

Here, we show results of top 1 and the best of top 5 predicted structures. The best performance is marked in bold font.

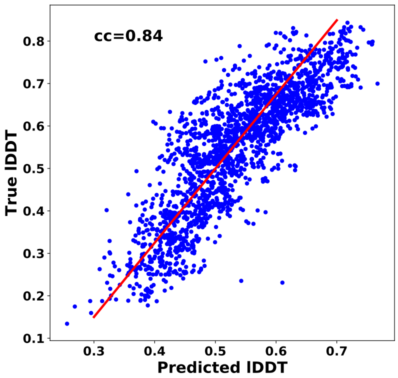

Figure S1: The correlation between the predicted lDDT score (x-axis) and the real lDDT score (y-axis) of the structure generated by FALCON2 on CASP13/14 target proteins

We also normalized the raw lDDT score into Z-score. As shown in Figure S2, when setting Z-score cut-off as 0.50, we obtained a total of 870 predicted structures for CASP13/14 target proteins, among which 770 predicted structures achieve TM-score over 0.40.

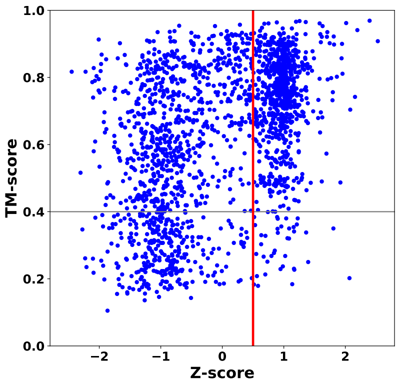

Figure S2: The relationship between Z-score and TM-score of predicted structures generated by FALCON2 on CASP13/14 target proteins

3 The effects of the availability of similar templates on the final prediction performance

To investigate prediction performance of FALCON2 server for proteins that have low similarity level with templates, we evaluate the correlation between the final prediction structure quality by FALCON2 and the similarity between a target protein and its optimal template. Here we used sequence identity as measure of similarity level. We used DeepAlign to find the optimal templated of 195 target proteins of CASP13 and CASP14.

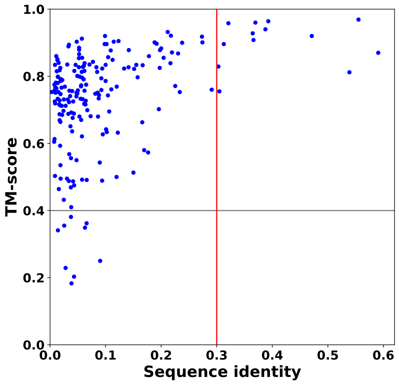

Figure S3: The effects of the availability of similar templates on the final prediction performance. Here, we calculate the sequence identity between a target protein and its most similar template as a measure of the availability of similar templates. Dataset: 195 CASP13/14 target proteins

As shown in Figure S3, 183 out of 195 target proteins have low sequence identity (less than 0.30) with most similar templates. Among these 183 target proteins, the predicted protein structure of 173 proteins has a TM-score over 0.4. The average TM-score of the predicted structure of 183 proteins is 0.723. These results demonstrate that FALCON2 can predict relatively high-quality protein structure for the target proteins with low similarity level with templates.

4 Performance of FALCON2 on the combined CASP13 and CASP14 datasets

In addition to evaluating FALCON2 on CASP13 and CASP14 datasets individually, we further evaluate FALCON2 on the combined CASP13 and CASP14 datasets that include a total of 195 target proteins.

As shown in Table xxx, the average TM-score of the top 1 predicted structure by FALCON2 is 0.735. Specifically, for the TBM-easy target, the average TM-score of the top 1 predicted structure by FALCON2 reaches 0.837. For the TBM-hard and TBM/FM targets, the average TM-score of the predicted top 1 structures is 0.732 and 0.722, respectively. Even for FM targets, the average TM-score of the predicted top 1 structures reaches 0.621.

We further analyzed the performance of ProFOLD and ProALIGN on this combined dataset. The average TM-score of the top 1 prediction by ProALIGN achieves 0.608, while it achieves 0.718 when using ProFOLD as predictor. By combining ProALIGN and ProFOLD, FALCON2 achieves higher structure prediction performance with an average TM-score of 0.735. These results demonstrate the power of the combination of the two approaches.

Table S3: The performance of FALCON2 on the combined CASP13 and CASP14 datasets (195 target proteins in total)

	All (195)	TBM-easy (66)	TBM-hard (49)	FM/TBM (27)	FM (54)
ProALIGN-only	0.608/0.624	0.811/0.827	0.655/0.672	0.507/0.529	0.365/0.379
ProFOLD-only	0.718/0.726	0.814/0.818	0.696/0.706	0.718/0.724	**0.621/0.631**
FALCON2	**0.735/0.745**	**0.837/0.848**	**0.732/0.741**	**0.722/0.726**	**0.621/0.631**

Here, we show results of top 1 and the best of top 5 predicted structures. The best performance is marked in bold font.

5 Two case studies: all-beta protein T0965 and alpha/beta protein T1026

Considering only two all-alpha proteins (T0966 and T0950) are discussed in the case studies section, here we show two more case studies: one is T0965, an alpha/beta protein, and the other is T1026, an all-beta protein.

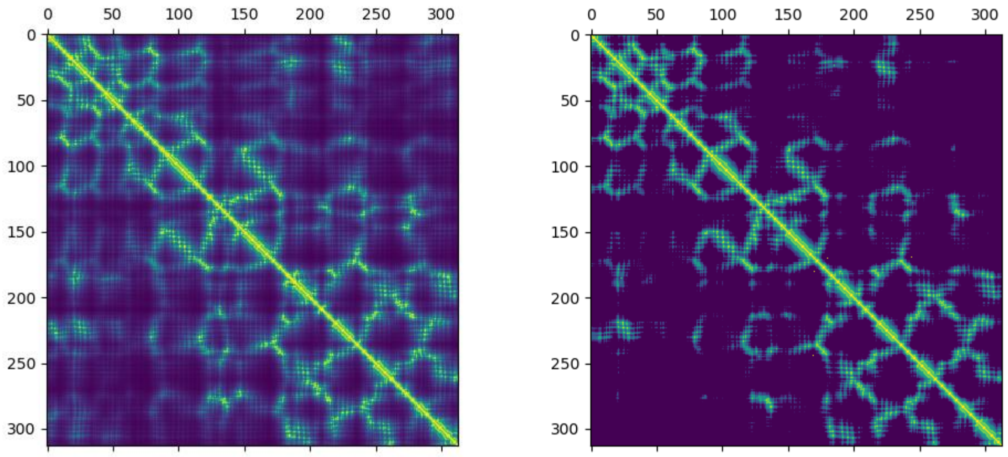

Figure S4: The native inter-residue distances (left panel) and the predicted inter-residue distances by ProFOLD (right panel) for target T0965

5.1 All-beta protein: CASP13 target T0965

Table S4: Precision of the inter-residue contacts predicted by ProFOLD for target T0965

	Top L	Top L/2	Top L/5	Top L/10
Long-range	0.891	0.987	1.000	1.000
Medium-range	0.300	0.564	0.903	1.000
Short-range	0.233	0.449	0.839	1.000

Here, we show the precision of the top L/10, L/5, L/2, and L residue contacts, where L represents protein length.

The target protein T0965 has a total of 334 residues, which was classified as TBM-hard in the CASP13 competition. T0965 is a chain of Apo Structure of TerB [2], and its native structure has already been solved and deposited in PDB as `6d2vA`.

Table S5: The top 5 templates reported by ProALIGN for target T0965

Template	Sequence Identity	CMO score	Template Quality	Predicted Model Quality
1sb8A	14.7%	0.718	0.813	0.832
1r6dA	19.2%	0.715	0.790	0.817
4zrmA	21.1%	0.715	0.810	0.831
1r66A	19.2%	0.713	0.791	0.811
2c5eA	19.2%	0.712	0.792	0.814

Here, we use TM-score to measure template quality and predicted structure quality.

Table S6: The top 5 predicted structures reported by ProQ3D for target T0965

Predicted structure	Predicted lDDT score	Z-Score	True lDDT score	TM-score of the predicted structure
T0965-PF-m1	0.585	1.467	0.655	0.878
T0965-PF-m2	0.583	1.359	0.655	0.875
T0965-PF-m3	0.569	0.763	0.643	0.868
T0965-PF-m4	0.567	0.686	0.648	0.877
T0965-PF-m5	0.558	0.315	0.653	0.877

Here, we use lDDT score and TM-score to measure predicted structure quality.

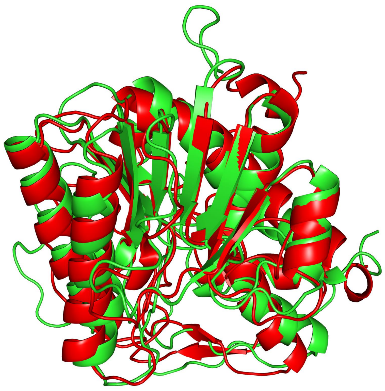

Figure S5: The native structure (in green) and predicted structure (in red) by FALCON2 for target T0965 (TM-score: 0.878)

For T0965, FALCON2 constructed an MSA through searching it against three sequence databases, including Uniclust30 (as of Oct. 2017), Uniref90 (as of Mar. 2018), and Metaclust (as of Jan. 2018). The constructed MSA contains a total of 63490 homologous proteins, implying that it may contain high-quality homology and co-evolution information. Next, FALCON2 executed ProFOLD to predict inter-residue distances for this target. Using the high-quality MSA, ProFOLD yielded accurate distance prediction. As shown in Table S4, the prediction accuracy of top L long-range residue contacts reaches 0.891. The predicted inter-residue distance matrix is also similar to the native

distance matrix(Fig. S4). Consequently, the predicted structure by ProFOLD achieved a TM-score of 0.878.

For this target, FALCON2 also executed ProALIGN to yield candidate structures. As shown in Table S5, ProALIGN reported 5 templates with high confidence score >0.7. Using predicted target-template alignments and related templates, ProALIGN yielded five high-quality structures with TM-score>0.8. For this target, Both ProFOLD and ProALIGN predicted high-quality structures with TM-score > 0.8, and finally, according to the predicted lDDT reported by ProQ3D (Table S6), FALCON2 selects a structure predicted by ProFOLD as the final prediction result (TM-score: 0.878; Fig. S5).

5.2 Alpha/beta protein: CASP14 target T1026

The target protein T1026 has a total of 172 residues, which was classified as TBM-hard in the CASP14 competition. T1026 is a chain of Faba bean necrotic stunt virus (FBNSV), and its native structure has already been solved and deposited in PDB as `6s44A`.

Table S7: Precision of the inter-residue contacts predicted by ProFOLD for target T1026

	Top L	Top L/2	Top L/5	Top L/10
Long-range	0.760	0.795	0.690	0.786
Medium-range	0.151	0.206	0.276	0.286
Short-range	0.171	0.233	0.379	0.571

Here, we show the precision of the top L/10, L/5, L/2, and L residue contacts, where L represents protein length.

Table S8: The top 5 templates reported by ProALIGN for target T1026

Template	Sequence Identity	CMO score	Template Quality	Predicted Model Quality
6f2sI	13.0%	0.537	0.699	0.742
4v4mh	5.5%	0.521	0.626	0.653
3s4gA	6.2%	0.519	0.645	0.666
4bcuA	5.5%	0.519	0.634	0.666
2bukA	5.5%	0.516	0.645	0.664

Here, we use TM-score to measure template quality and predicted structure quality.

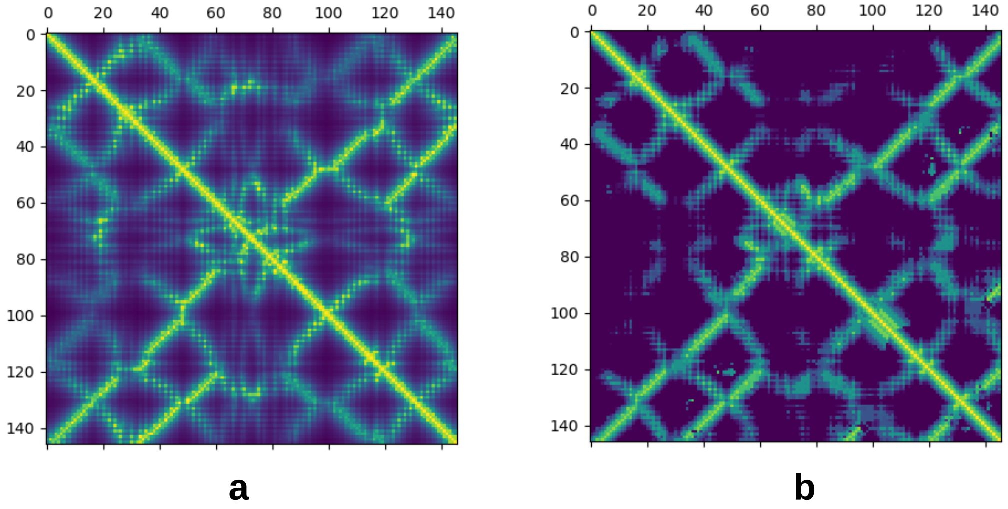

Figure S6: The native inter-residue distances (left panel) and the predicted inter-residue distances by ProFOLD (right panel) for target T1026

Table S9: The top 5 predicted structures reported by ProQ3D for target T1026

Predicted structure	Predicted lDDT score	Z-Score	True lDDT score	TM-score of the predicted structure
T1026-PA-6f2sI	0.607	2.158	0.535	0.742
T1026-PA-4bcuA	0.524	0.715	0.461	0.666
T1026-PA-4v4mh	0.516	0.573	0.455	0.653
T1026-PA-3s4gA	0.512	0.508	0.462	0.666
T1026-PA-2bukA	0.495	0.211	0.458	0.664

Here, we use lDDT score and TM-score to measure predicted structure quality.

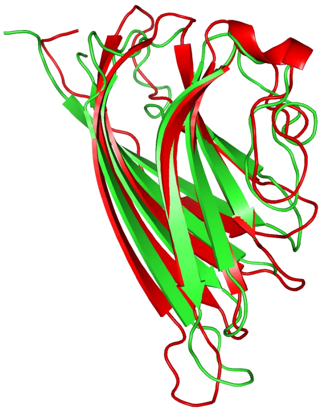

Figure S7: The native structure (in green) and predicted structure (in red) by FALCON2 for target T1026 (TM-score: 0.742)

For T1026, FALCON2 constructed an MSA through searching it against four sequence databases, including Uniref30 (as of Feb. 2020), Uniref90 (as of Feb. 2020), BFD (as of Mar. 2019), and MGnify90 (as of May. 2019). The constructed MSA contains a total of 20 homologous proteins, implying that its quality is relatively lower. Next, FALCON2 executed ProFOLD to predict inter-residue distances for this target. Although the MSA used has lower quality, ProFOLD still yielded relatively high-quality distance prediction. As shown in Table S7, the prediction accuracy of top L long-range residue contacts is 0.760. Consequently, the predicted structure by ProFOLD achieved a TM-score of 0.609.

For this target, FALCON2 also executed ProALIGN to yield candidate structures. As shown in Table S8, ProALIGN reported 5 templates with confidence score >0.5. Using predicted target-template alignments and selected templates, ProALIGN yielded five structures with TM-score>0.6. Among them, the TM-score of the best structure was 0.742. Finally, according to the predicted lDDT reported by ProQ3D (Table S9), FALCON2 selected a structure predicted by ProALIGN as the final prediction result (TM-score: 0.742; Fig. S7).

6 The user interface of FALCON2 server and usage explanation

6.1 The job submission interface of FALCON2 server

Figure S8 shows the job submission of FALCON2 server. An end-user can import a sequence file (in FASTA format) or simply paste the target protein sequences into the text box. Users can define a job ID for the job; otherwise, FALCON2 will automatically assign one. Filling email address is optional and if it was filled, the prediction results will be sent to the email address.

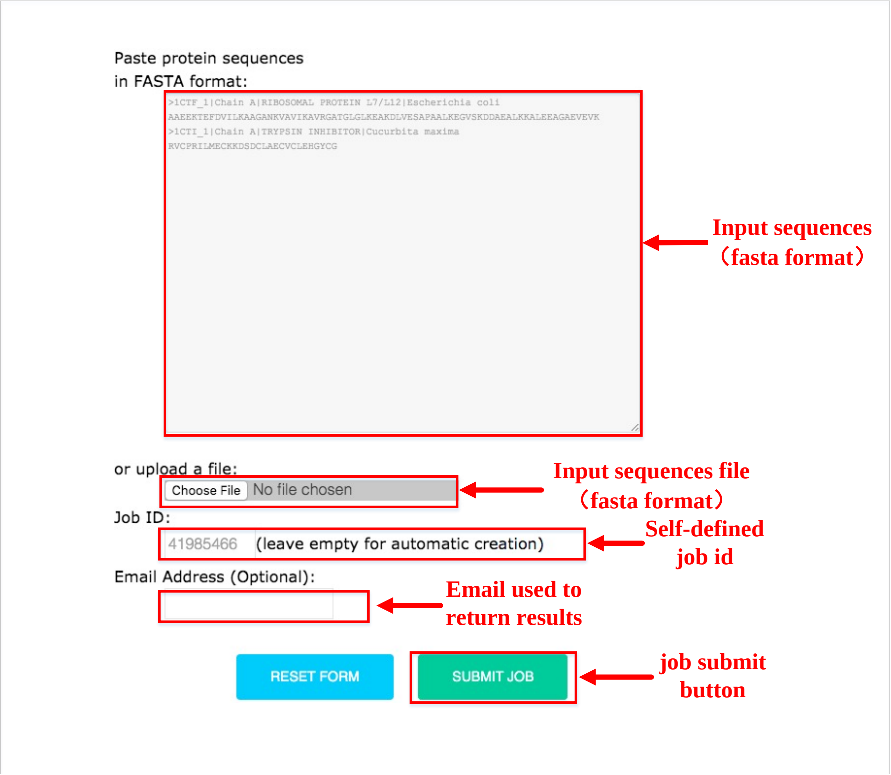

Figure S8: The job submission interface of FALCON2 server

6.2 The running status interface of FALCON2 server

When receiving a target protein sequence, FALCON2 will automatically jump to the running status interface. As the job might contain multiple target proteins, FALCON2 allows checking status of each individual target protein (Fig. S9).

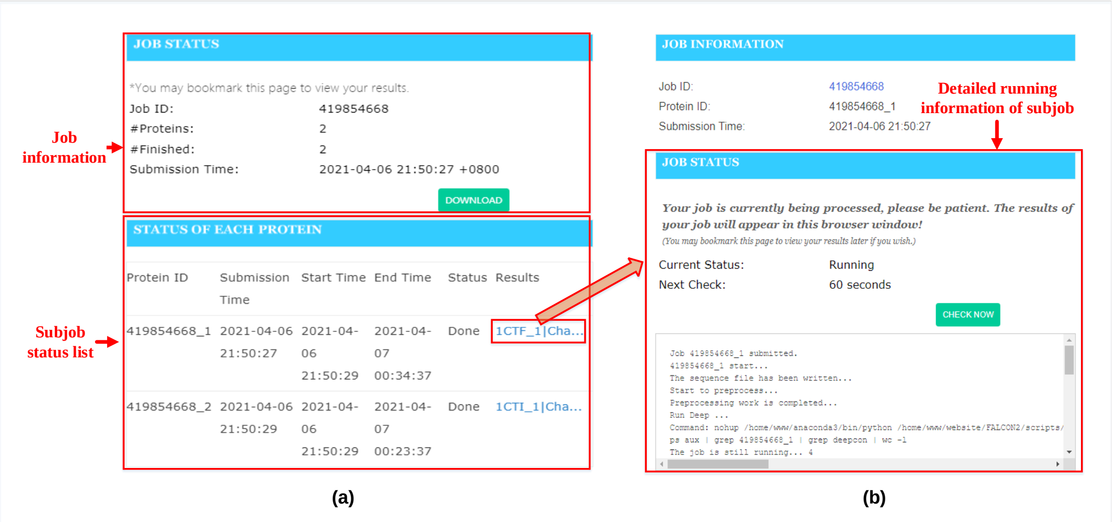

Figure S9: The running status interface of FALCON2 server

6.3 Result display interface of FALCON2 server

When the prediction job is finished, the job status will be updated. By clicking the URL shown in the status page, users can check the final prediction results (Fig. S10). Besides the final predicted structures (Fig. S11), the result display interface also shows some intermediate results, including the target-template alignments predicted by ProALIGN (Fig. S12 and Fig. S14), and the inter-residue contacts predicted by ProFOLD (Fig. S13). FALCON2 uses 3Dmol[3] package to show the predicted 3D structures, thus allowing free rotation and zooming in/out of structures. This page also provides a link for downloading all prediction results, including the MSA for target proteins, the predicted inter-residue distances, target-template alignments, and the constructed structures.

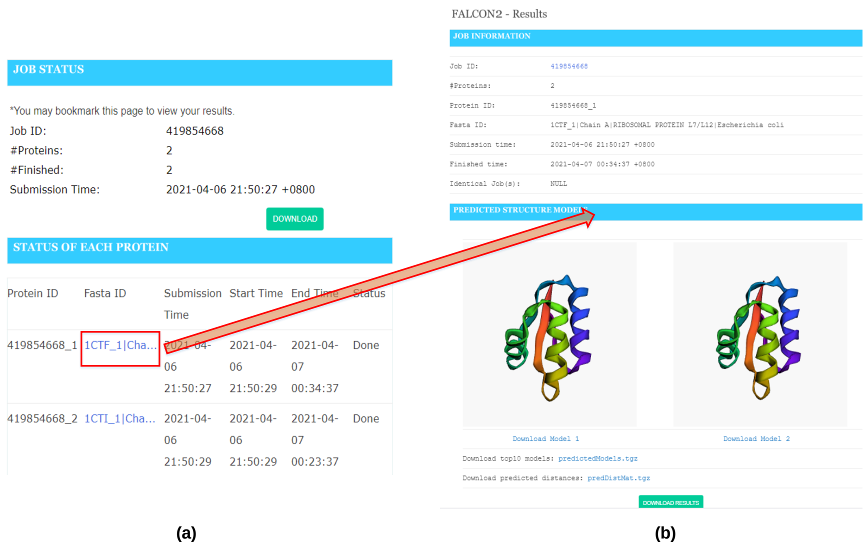

Figure S10: The updated job status interface when the prediction job is finished

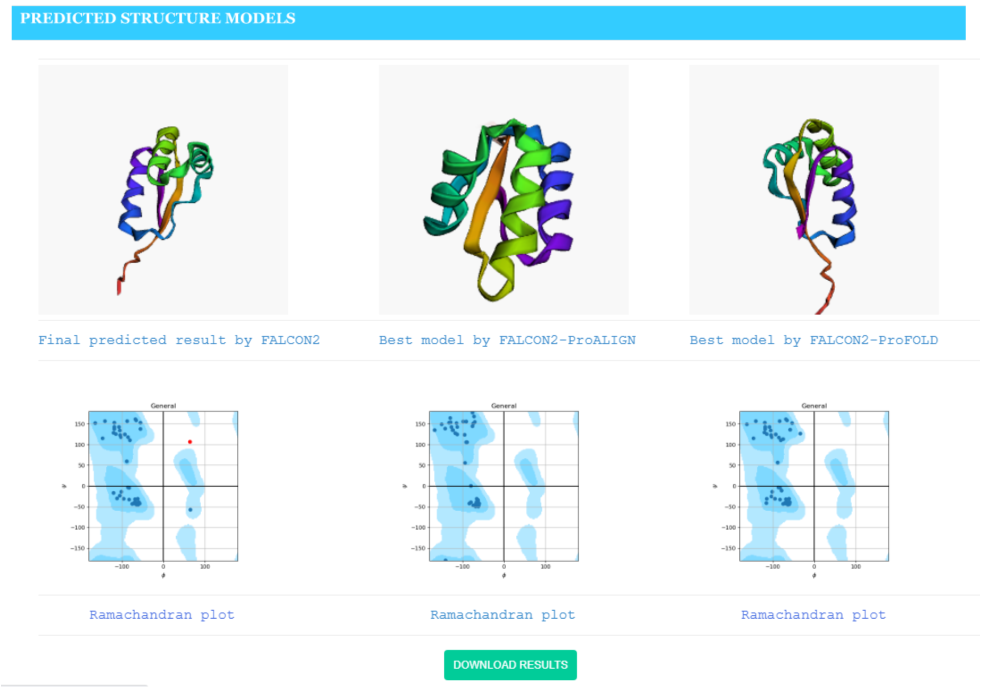

Figure S11: The final predicted structure by FALCON2. Besides the structure selected by ProQ3D, FALCON2 also shows the best structure predicted by ProALIGN and ProFOLD

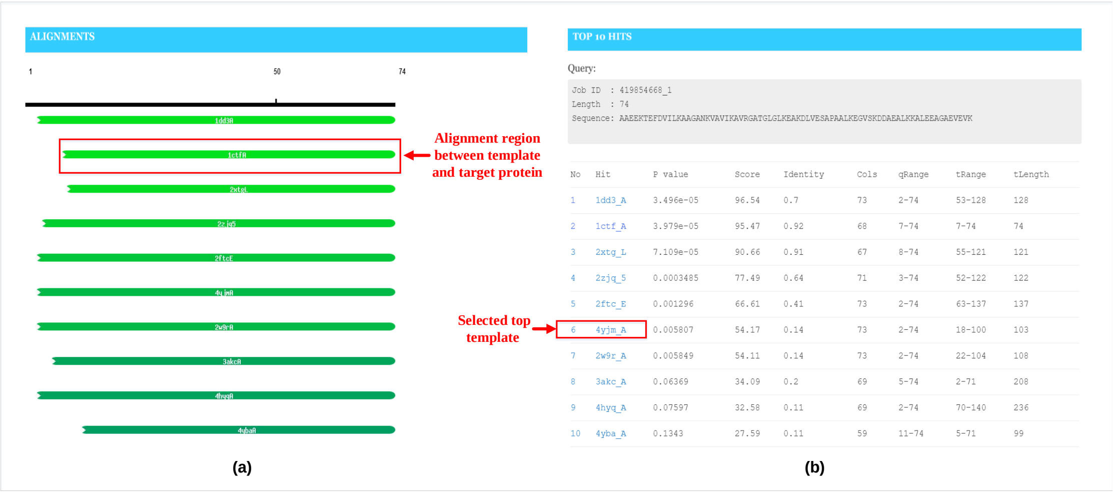

Figure S12: The top templates reported by ProALIGN

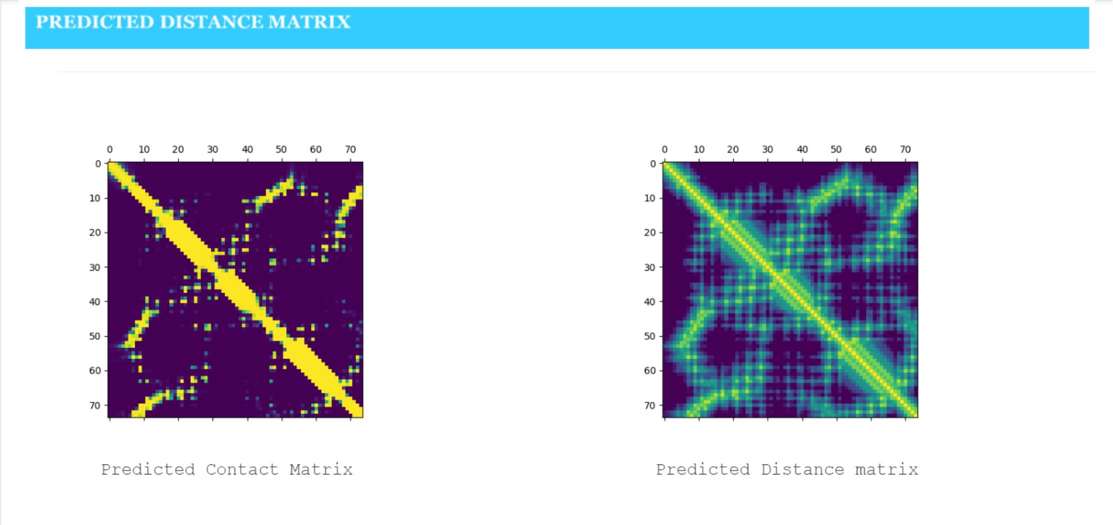

Figure S13: The predicted inter-residue contacts and distances by ProFOLD

THE DETAIL FOR ALIGNMENT

No 1: Template 1ctf_A

Annotation: Ribosomal protein L7/L12; 1.70A {Escherichia coli} SCOP: d.45.1.1 PDB: 1rqs_A 2bcw_B

Built Model:

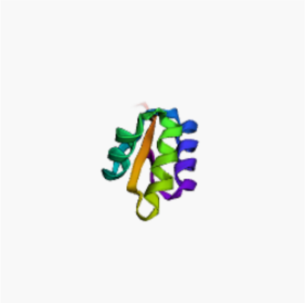

S	acc_conf	1	44486845477665597749693494569695759895554669865675875768798847777449	68 (68)
S	acc_pred	1	MBEBMBMEMEEEEEMBBEMBMEBMEMEMEEBMEMBEEMEMMBEEEMMEEMBEEBMEMBEEMEBEBEME	68 (68)
S	sse_conf	1	44589966799969999999995799999999999669966755999999999999999879978878	68 (68)
S	sse_pred	1	CCEEEEEECCCCHHHHHHHHHHHCCCCHHHHHHHHHCCCHHHHCCCCHHHHHHHHHHHHHCCCEEEEC	68 (68)
S	1ctfA_2021_1	1	EFDVILKAAGANKVAVIKAVRGATGLGLKEAKDLVESAPAALKEGVSKDDAEALKKALEEAGAEVEVK	68 (68)
T	1ctf_A	1	EFDVILKAAGANKVAVIKAVRGATGLGLKEAKDLVESAPAALKEGVSKDDAEALKKALEEAGAEVEVK	68 (68)
T	sse_real	1	CEEEEEEECCCCHHHHHHHHHHHHCCCHHHHHHHHHCCCEEEEEEECHHHHHHHHHHHHHHCCEEEEC	68 (68)
T	acc_real	1	EMMBMBMMBEEEMEEBBEMBEEEMEMMEEMBMEMBEEMEMEMEEEMEEEEBMEMMEMBEEMMBEMMME	68 (68)

Figure S14: The target-template alignment predicted by ProALIGN

References

[1] B. Bräuning, E. Bertosin, F. Praetorius, C. Ihling, A. Schatt, A. Adler, K. Richter, A. Sinz, H. Dietz, and M. Groll. Structure and mechanism of the two-component α-helical pore-forming toxin YaxAB. *Nature Communications*, 9(1):1–14, 2018.

[2] J. A. Clinger. *Structure, Function, and Dynamics of Bacterial Phytochromes and Natural Product Biosynthesis Enzymes*. PhD thesis, Rice University, 2018.

[3] N. Rego and D. Koes. 3Dmol. js: molecular visualization with WebGL. *Bioinformatics*, 31(8):1322–1324, 2015.

[4] J. Xu and Y. Zhang. How significant is a protein structure similarity with TM-score= 0.5? *Bioinformatics*, 26(7):889–895, 2010.
